# Cell Wall Ultrastructure of Stem Wood, Roots, and Needles of a Conifer Varies in Response to Moisture Availability

**DOI:** 10.3389/fpls.2016.00882

**Published:** 2016-06-24

**Authors:** Sivakumar Pattathil, Miles W. Ingwers, Olivia L. Victoriano, Sindhu Kandemkavil, Mary Anne McGuire, Robert O. Teskey, Doug P. Aubrey

**Affiliations:** ^1^Complex Carbohydrate Research Center, University of GeorgiaAthens, GA, USA; ^2^Daniel B. Warnell School of Forestry and Natural Resources, University of GeorgiaAthens, GA, USA; ^3^Savannah River Ecology Laboratory, University of GeorgiaAiken, SC, USA

**Keywords:** cell walls, glycome profiling, moisture stress, monoclonal antibodies, pectin, *Pinus taeda*, xylan

## Abstract

The composition, integrity, and architecture of the macromolecular matrix of cell walls, collectively referred to as cell wall ultrastructure, exhibits variation across species and organs and among cell types within organs. Indirect approaches have suggested that modifications to cell wall ultrastructure occur in response to abiotic stress; however, modifications have not been directly observed. Glycome profiling was used to study cell wall ultrastructure by examining variation in composition and extractability of non-cellulosic glycans in cell walls of stem wood, roots, and needles of loblolly pine saplings exposed to high and low soil moisture. Soil moisture influenced physiological processes and the overall composition and extractability of cell wall components differed as a function of soil moisture treatments. The strongest response of cell wall ultrastructure to soil moisture was increased extractability of pectic backbone epitopes in the low soil moisture treatment. The higher abundance of these pectic backbone epitopes in the oxalate extract indicate that the loosening of cell wall pectic components could be associated with the release of pectic signals as a stress response. The increased extractability of pectic backbone epitopes in response to low soil moisture availability was more pronounced in stem wood than in roots or needles. Additional responses to low soil moisture availability were observed in lignin-associated carbohydrates released in chlorite extracts of stem wood, including an increased abundance of pectic arabinogalactan epitopes. Overall, these results indicate that cell walls of loblolly pine organs undergo changes in their ultrastructural composition and extractability as a response to soil moisture availability and that cell walls of the stem wood are more responsive to low soil moisture availability compared to cell walls of roots and needles. To our knowledge, this is the first direct evidence, delineated by glycomic analyses, that abiotic stress affects cell wall ultrastructure. This study is also unique in that glycome profiling of pine needles has never before been reported.

## Introduction

The cell wall is the main determinant of form, structure, and mechanical strength of plant cells and ultimately influences cell function (Cosgrove, 1997). The composition, integrity, and architecture of the macromolecular matrix of cell walls, collectively referred to as ultrastructure (Pattathil et al., 2015), varies across plant systematic classes (Popper et al., 2011; Fangel et al., 2012), across organs within a species (Moller et al., 2007), and among cell types within an organ (Freshour et al., 1996; Knox, 2008; Popper et al., 2011). Plant cell walls can also be dynamic through time, routinely exhibiting changes in structure and composition throughout development (Freshour et al., 1996; Moller et al., 2007). Moreover, changes to cell wall structure and composition may also occur sporadically as a plant experiences various abiotic or biotic stresses (Tenhaken, 2015).

A variety of approaches have suggested that adjustments to plant cell wall ultrastructure occur in response to abiotic stress. Most studies investigating the effects of abiotic stress on cell wall dynamics have focused on the genes believed to be responsible for cell wall metabolism (Tenhaken, 2015). Recent investigations have explored differences in protein expression in cell walls of stressed and control plants through proteomic profiling (Dani et al., 2005; Zhu et al., 2007; Kong et al., 2010). Although these approaches have provided some insight into the response of plant cell walls to abiotic stress, they do not provide direct information about cell wall ultrastructure. Glycome profiling offers a powerful and novel way to investigate modifications to cell wall ultrastructure in response to abiotic stress by directly examining changes in the composition, integrity, and architecture of cell walls.

Drought is an important abiotic stress that influences plant growth and production; however, we are unaware of any studies that have directly investigated differences in the composition, integrity, and architecture of cell walls in response to soil moisture availability using a glycomic approach. A recent literature review, based on the indirect approaches discussed above, suggested that the biosynthesis of lignin, hemicellulose, and pectic side chains would increase under moisture stress (Le Gall et al., 2015).

Here, we employed glycome profiling to elucidate differences in cell wall ultrastructure by examining variation in the composition and extractability of major non-cellulosic cell wall glycans in response to high (−0.3 MPa) and low (−1.5 MPa) soil moisture treatments among stem wood, roots, and needles of loblolly pine saplings. We hypothesized that cell wall ultrastructure would differ among organs and would respond to differences in soil moisture. Specifically, we predicted that low soil moisture would enhance the extractability of hemicellulose and pectic components and elicit changes in lignin–glycan associations.

## Materials and methods

### Plant material

Two-year-old sapling-stage ramets of one loblolly pine (*Pinus taeda* L.) clone (Arborgen, Summerville, Inc., SC, USA) were planted in 4.3 L tree pots (CP612R, Steuwe and Son, Inc., Tangent, OR USA) in a mixture of fritted clay (Turface Field and Fairway, Profile Products LLC, Buffalo, IL, USA) and fine sand (3:1, v:v) in late March 2013. Saplings were grown in a greenhouse at the Whitehall Experimental Forest in Athens, GA, USA, under air temperature that approximately matched ambient and high relative humidity. Each sapling received 40 g of 15–9–12 extended release fertilizer (Osmocote Plus #903286, Scotts-Sierra Horticultural Products, Marysville, Ohio, USA) and 0.2 g of chelated iron (Sprint 138, Becker Underwood, Ames, Iowa USA) as a single amendment 2 weeks after planting. Saplings were grown under well-watered conditions for ~11 weeks prior to initiating soil moisture treatments.

### Experimental design and treatments

A total of 114 saplings were randomly assigned to either a high or low soil moisture treatment. These saplings were arranged in three blocks (*n* = 3) that consisted of one replicate plot of each treatment, with 19 saplings per plot. Soil moisture treatments were maintained at constant soil volumetric water content that coincided with soil water potential of −0.3 MPa in the high soil moisture treatment and −1.5 MPa in the low soil moisture treatment. Treatments were applied using an irrigation system (Nemali and van Iersel, 2006) consisting of soil moisture sensors (EC-5 sensor, Decagon Devices, Pullman, WA USA), a datalogger (CR23X, Campbell Scientific, Logan, Utah, USA), and solenoids (Model 57100, Orbit, North Salt Lake, Utah, USA) to precisely control soil moisture. Saplings were grown under soil moisture treatment conditions for a 12-week period from June 15, 2013 to September 7, 2013.

### Growth and physiological measurements

Midday (14:00–16:00) leaf gas exchange measurements, including net assimilation (*A*_net_, μmol CO_2_ m^−2^ s^−1^), transpiration (*E*, mol H_2_O m^−2^ s^−1^), and stomatal conductance (*g*_s_, mol H_2_O m^−2^ s^−1^) were made on a single fascicle of a randomly selected sapling in each plot during the final week of the experiment prior to harvest. Measurements were made with a portable photosynthesis system (LI-6400, Li-Cor Biosciences, Lincoln, NE, USA) fitted with a standard leaf chamber. Chamber conditions were set to 1000 μmol m^−2^ s^−1^ PAR (Photosynthetically Active Radiation) and 30°C block temperature. Chamber humidity was adjusted to approximately match ambient. Needle diameter was recorded at the time of measurement to calculate total needle surface area enclosed in the chamber. Pre-dawn and afternoon needle xylem pressure potential, which we define as needle water potential, were measured in conjunction with gas exchange measurements using a pressure chamber (Model 600, PMS Instruments Co., Corvallis, Oregon, USA).

Biomass accumulation was determined as the difference in biomass between harvests at the beginning and end of the experiment. Four saplings in each treatment replicate were randomly selected for each harvest. Height and diameter were recorded for each harvested sapling and their stems, roots, and needles were separated and dried at 65°C to a constant mass to determine total biomass.

At final harvest, needle tissue samples from the most recent flush of each of the four harvested saplings per treatment replicate were analyzed for stable carbon isotope ratio (δ^13^C) to provide a time-integrative measure of soil moisture stress (Farquhar et al., 1982, 1989). Stable isotope analysis was performed at the Stable Isotope and Soil Biology Laboratory, Odum School of Ecology, University of Georgia, Athens, GA, USA.

### Glycome profiling analyses

Glycome profiling was conducted at the Complex Carbohydrate Research Center (CCRC), University of Georgia, Athens, GA, USA. Glycome profiling analyses were used to determine how composition and extractability of major non-cellulosic cell wall glycans varied in response to soil moisture treatments in stem wood, roots, and needles of loblolly pine saplings. Glycome profiles of cell walls were analyzed for each organ × treatment combination using the techniques described in Pattathil et al. (2012) and DeMartini et al. (2011). Briefly, tissue samples from three randomly-selected saplings per treatment were used. Needle and stem wood tissue that developed after treatments were imposed was harvested from each sapling. For each organ at least 5 g of material was used. For stem wood samples, the outer bark and vascular cambium were removed, so that only wood was included in the analysis. Root and needle samples were fully intact and contained all associated tissues. As it was impossible to determine if a particular root developed completely under treatment, we used only the distal 4 cm of any given root. Although we could not determine with absolute certainty that root tissue developed under treatment conditions, we considered this to be an adequate approach. Tissue samples were ground, dried, and extracted in alcohol to remove alcohol-soluble compounds. Glycome profiles were constructed for the remaining alcohol-insoluble residues (AIR). The first step of glycome profiling consisted of sequential extraction of cell wall materials from the AIR using increasingly harsh reagents. This approach allowed selective enrichment of various cell wall glycans in the extracts on the basis of the relative tightness with which they were integrated into the walls (Pattathil et al., 2012). For instance, oxalate and carbonate extractions release loosely bound pectic polysaccharides; 1 and 4 M KOH extractions isolate hemicelluloses, more tightly integrated pectic polysaccharides, and chlorite extraction fragments, and remove lignin releasing lignin associated polysaccharides; and the final 4 M KOHPC extraction step recovers the remaining hemicelluloses and pectins that are otherwise integrated to the wall indirectly or directly through association/s with lignin. The resulting extracts were then screened with a comprehensive suite of 155 cell wall glycan-directed monoclonal antibodies (mAbs) specific to glycan epitopes present in most major non-cellulosic carbohydrate components of plant cell walls. Carbohydrate extracts were diluted to 20 μg glucose equivalent per ml solutions and were loaded into a 384-well enzyme-linked immunosorbent assay (ELISA) plate (Costar 3700, Corning Inc., Corning, NY, USA) at 15 μl per well. ELISA analyses were run using a 384 well ELISA Robotic System (Thermo Fisher Scientific Inc. Waltham, MA, USA). The binding strengths of mAbs correspond directly to the abundance of the specific glycan epitope structures. Comparison of glycan extractability and mAbs binding strength across the three organs (stem wood, roots, and needles) and within organs as a function of soil moisture facilitated detailed comprehension of variation in cell wall ultrastructure.

The plant cell wall glycan-directed mAbs employed in glycome profiling were obtained from laboratory stocks (CCRC, JIM, and MAC series) and are available through CarboSource Services (http://www.carbosource.net) or BioSupplies (Australia; BG1, LAMP). More detailed descriptions of these mAbs are available at the web database, WallMAbDB (http://www.wallmabdb.net) and are also provided in an interactive table (Supplementary Table 1).

### Statistical analysis

Physiological and growth measurements were analyzed using a mixed model approach (PROC MIXED; Version 9.3, SAS Inc., Cary, NC, USA; Littell et al., 1996). Soil moisture treatments were applied at the plot level; therefore, the experimental unit was the plot and consisted of the mean value of individual saplings measured within that plot. The experimental unit (plot) was treated as a random factor (*n* = 3 per treatment) and soil moisture treatment was treated as a fixed factor (*n* = 2). In the glycome profiling data, differences in epitope binding were assessed with an ANOVA (JMP Genomics, SAS Inc., Cary, NC, USA) using the method described in Liang et al. (2013). We used a type-I error rate of 0.05 and present percent differences as a measure of effect size.

## Results

### Physiology and growth

The main purpose of the physiological and growth measurements was to demonstrate instantaneous and cumulative responses to soil moisture treatments. We observed lower rates of gas exchange and lower needle water potential in the low compared to the high soil moisture treatment (Table 1), which indicates that the low soil moisture treatment elicited physiological stress responses. For example, pre-dawn needle water potential was −1.93 MPa in the low compared to −0.58 MPa in the high soil moisture treatment (*p* = 0.0155). Likewise, *A*_*net*_ and *E* were 94 and 84% lower in the low compared to the high soil moisture treatment (*p* = 0.0019 and *p* = 0.0492, respectively). Although stomatal conductance (*g*_*s*_) was 0.009 ± 0.003 mol H_2_O m^−2^ s^−1^ in the low and 0.072 ± 0.026 mol H_2_O m^−2^ s^−1^ in the high soil moisture treatment, the difference was not significant (*p* = 0.0793).

**Table 1:** **Mean (standard error) predawn water potential, afternoon water potential and midday instantaneous gas exchange (14:00–16:00) of loblolly pine saplings grown at high (−0.3 MPa) and low (−1.5 MPa) soil moisture**.

	**−0.3 MPa**	**−1.5 MPa**	***p*-values**
Afternoon pressure potential (MPa)	−1.07(0.12)	−2.20(0.26)	**0.0159**
Predawn pressure potential (MPa)	−0.58(0.10)	−1.93(0.32)	**0.0155**
*A_net_* (μmol CO_2_ m^−2^ s^−1^)	6.24(0.75)	0.35(0.32)	**0.0019**
*E* (mmol H_2_O m^−2^ s^−1^)	2.10(0.63)	0.33(0.9)	**0.0492**
*gs*(mol H_2_0 m^−2^ s^−1^)	0.072(0.026)	0.009(0.003)	0.0793

*Gas exchange parameters include net assimilation (A_net_), transpiration (E), and stomatal conductance (g_s_). Significant differences at α = 0.05 are shown in bold*.

**Figure 1:**
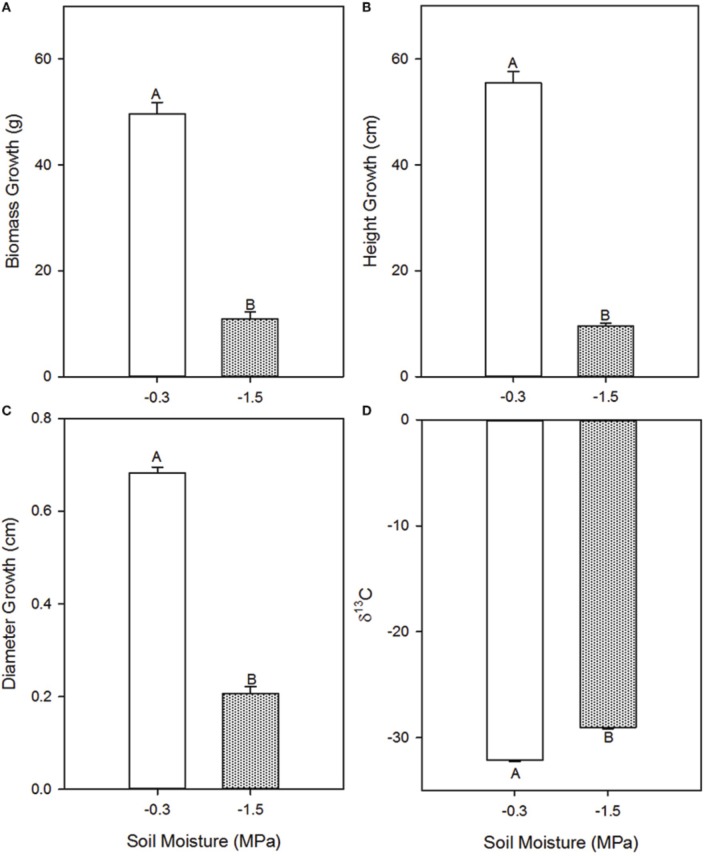
**Effects of high (−0.3 MPa) and low (−1.5 MPa) soil moisture treatments on growth and δ^**13**^C of ***Pinus taeda*** saplings**. Mean (*SE*) biomass accumulation **(A)**, height growth **(B)**, diameter growth **(C)**, and needle δ^13^C **(D)** of loblolly pine saplings grown for 12 weeks at high and low soil moisture. Thin bars represent standard error. Letters denote statistical significance at α = 0.05.

Growth parameters were consistently lower in the low compared to the high soil moisture treatment (Figure 1), indicating that the treatments induced a cumulative effect on development. Total biomass accumulation was 78% lower (*p* = 0.0011), height was 83% lower (*p* = 0.0001), and stem diameter was 67% lower (*p* < 0.0001) in the low compared to the high soil moisture treatment. Carbon isotope discrimination (δ^13^C), a time-integrative measurement of water stress (Farquhar et al., 1982, 1989), was −29.04 in the low soil moisture treatment and −32.163 in the high soil moisture treatment (*p* < 0.0001), indicating that observed differences in instantaneous rates of gas exchange persisted through time (Figure 1).

### Glycome profiling data

**Figure 2:**
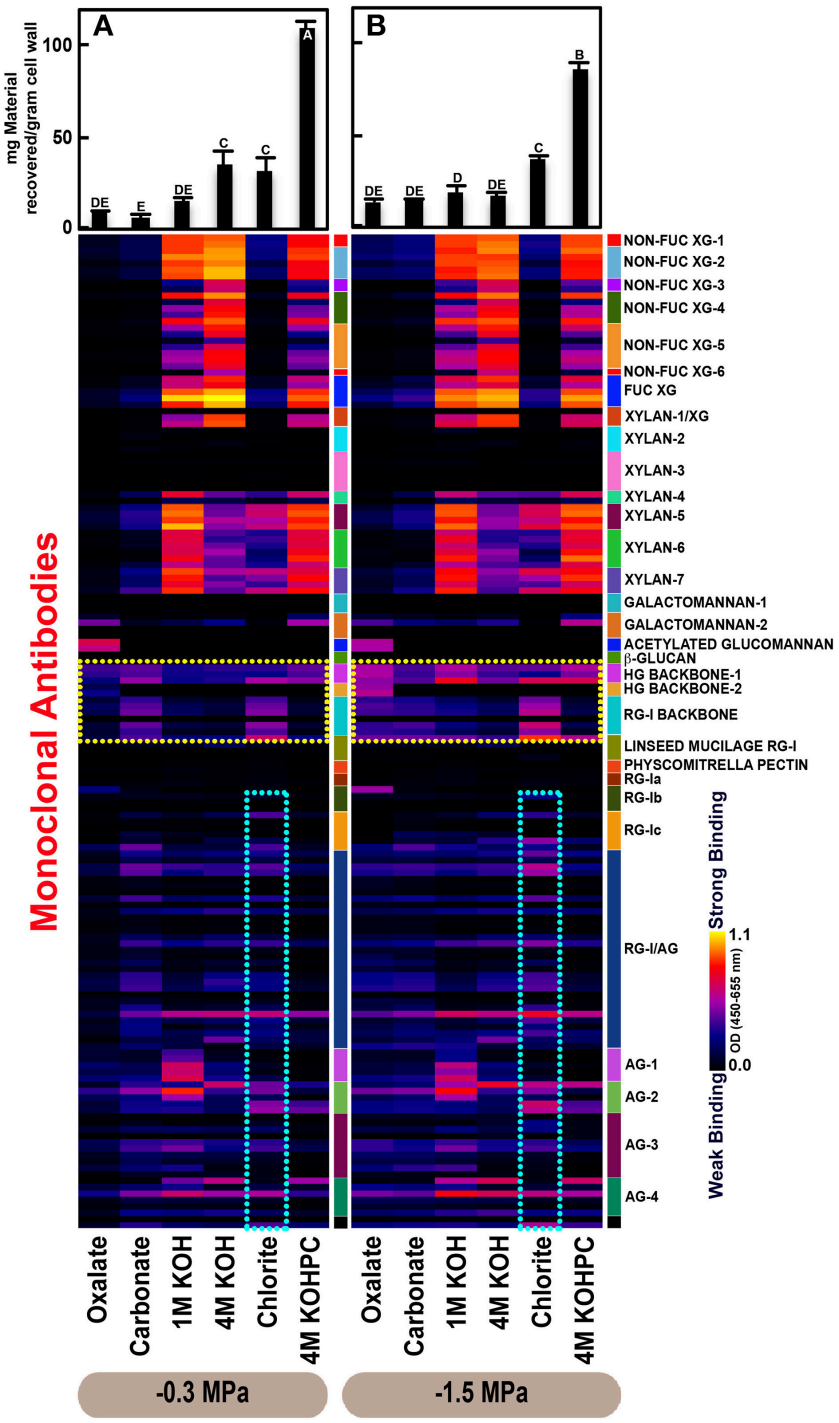
**Glycome profiles of cell walls isolated from stem wood of ***Pinus taeda*** saplings subjected to high (A; −0.3 MPa) and low (B;−1.5 MPa) soil moisture treatments**. Cell wall materials (alcohol insoluble residues, AIR) were isolated from stem wood of loblolly pine saplings. Sequential extracts were prepared from cell wall materials using increasingly harsh reagents (from oxalate to 4 M KOHPC) to facilitate the selective extraction of glycans based on the relative tightness with which they were integrated into the cell wall. The extracts were then ELISA screened with a comprehensive collection of 155 glycan directed mAbs that are specific to most major non-cellulosic cell wall glycans (panel on right denotes specific glycan groups recognized by mAbs). The strength of binding of the mAbs is depicted as a heatmap with bright yellow depicting the strongest binding, dark blue, no binding, and red, intermediate binding. The binding strength of each antibody directly corresponds to the abundance of the specific glycan epitope structure it recognizes. Amount of material recovered (mg/g AIR) from each sequential extraction shown at the top of each panel. Data are the mean of three biological replicates. Thin bars represent standard error. Letters denote statistical significance at α = 0.05.

**Figure 3:**
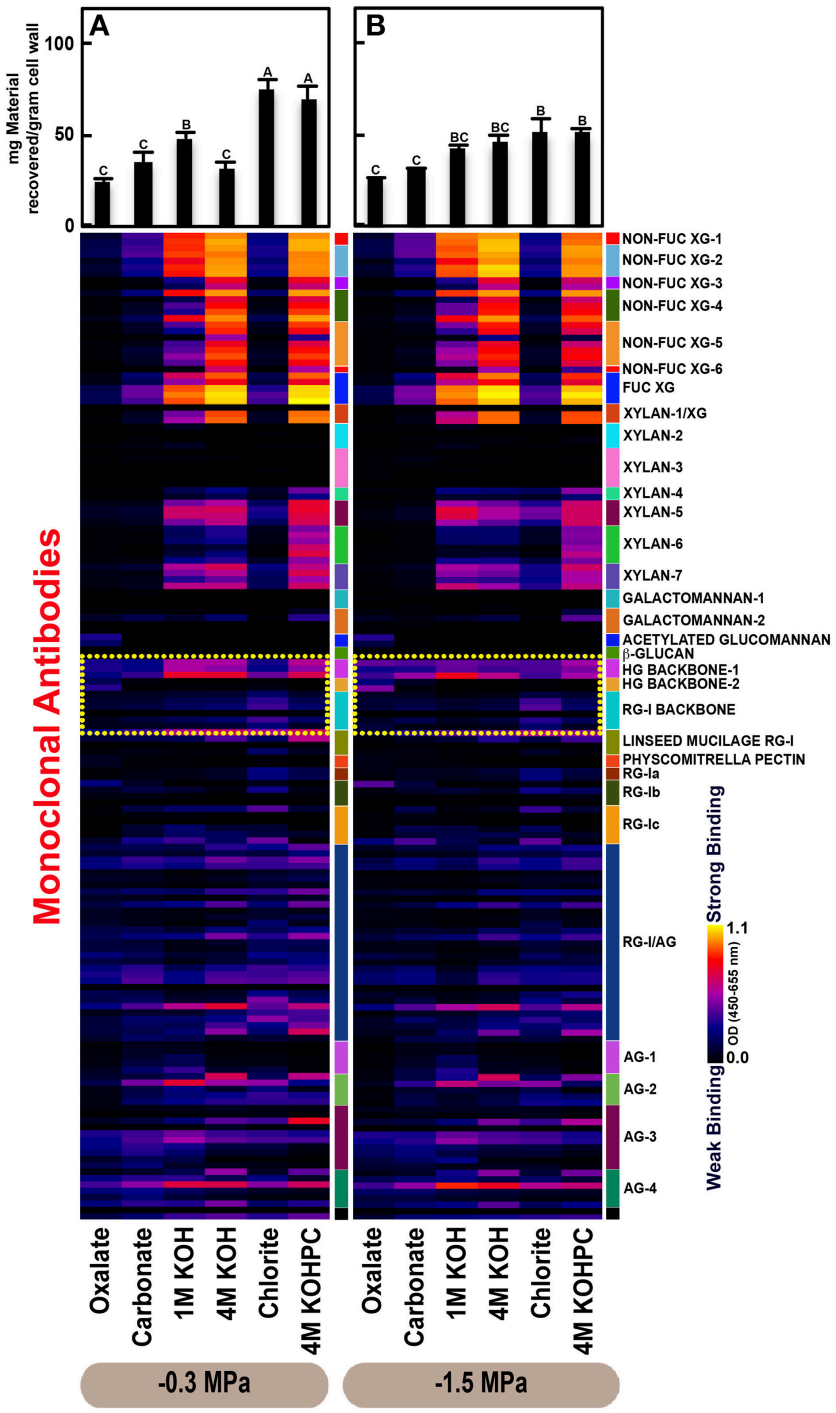
**Glycome profiles of cell walls isolated from roots of ***Pinus taeda*** saplings subjected to high (A;−0.3 MPa) and low (B;−1.5 MPa) soil moisture treatments**. Cell wall materials (alcohol insoluble residues, AIR) were isolated from roots of loblolly pine saplings. Sequential extracts were prepared from cell wall materials using increasingly harsh reagents (from oxalate to 4 M KOHPC) to facilitate the selective extraction of glycans based on the relative tightness with which they were integrated into the cell wall. The extracts were then ELISA screened with a comprehensive collection of 155 glycan directed mAbs that are specific to most major non-cellulosic cell wall glycans (panel on right denotes specific glycan groups recognized by mAbs). The strength of binding of the mAbs is depicted as a heatmap with bright yellow depicting the strongest binding, dark blue, no binding, and red, intermediate binding. The binding strength of each antibody directly corresponds to the abundance of the specific glycan epitope structure it recognizes. Amount of material recovered (mg/g AIR) from each sequential extraction shown at the top of each panel. Data are the mean of three biological replicates. Thin bars represent standard error. Letters denote statistical significance at α = 0.05.

**Figure 4:**
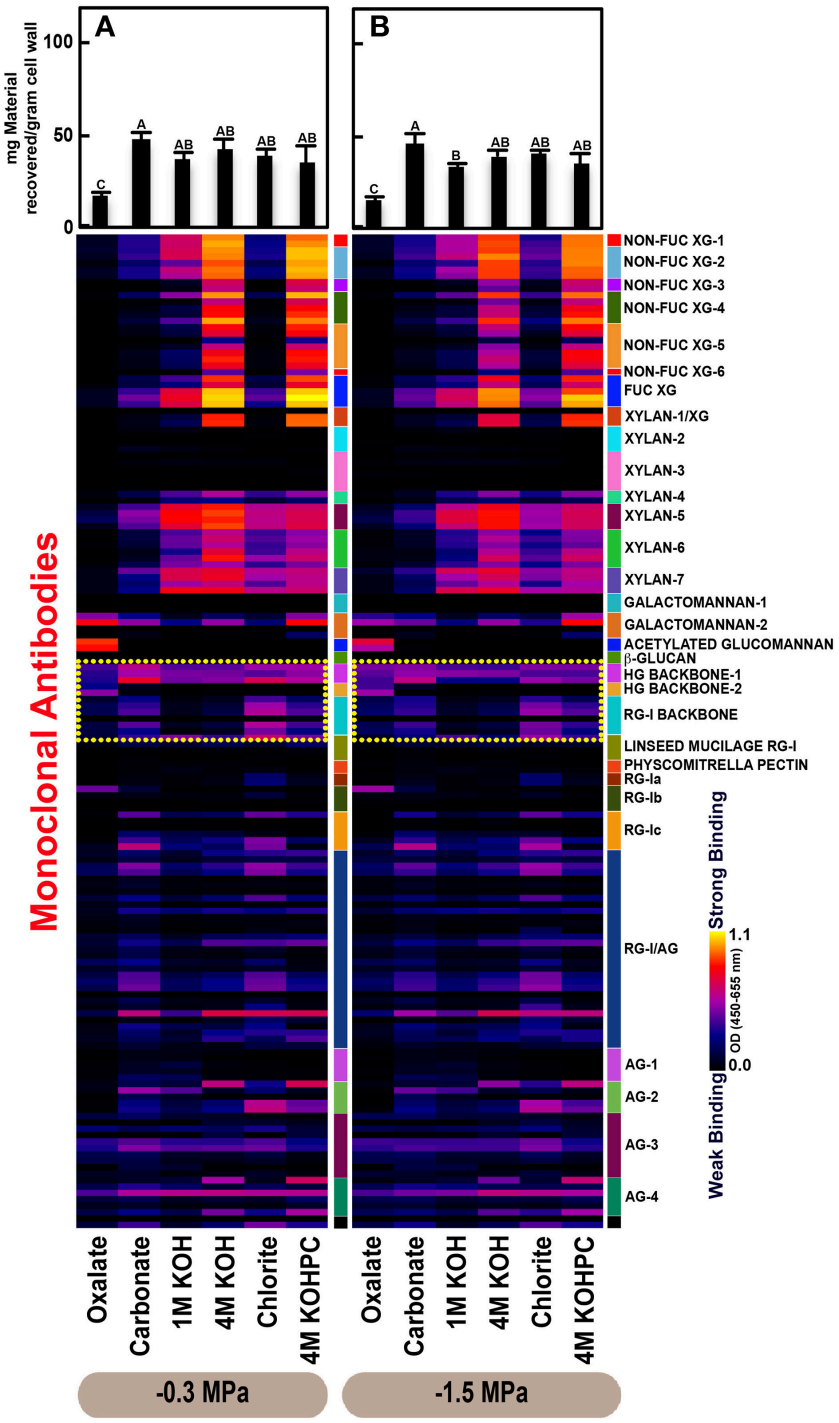
**Glycome profiles of cell walls isolated from needles of ***Pinus taeda*** saplings subjected to high (A; −0.3 MPa) and low (B; −1.5 MPa) soil moisture treatments**. Cell wall materials (alcohol insoluble residues, AIR) were isolated from needles of loblolly pine saplings. Sequential extracts were prepared from cell wall materials using increasingly harsh reagents (from oxalate to 4 M KOHPC) to facilitate the selective extraction of glycans based on the relative tightness with which they were integrated into the cell wall. The extracts were then ELISA screened with a comprehensive collection of 155 glycan directed mAbs that are specific to most major non-cellulosic cell wall glycans (panel on right denotes specific glycan groups recognized by mAbs). The strength of binding of the mAbs is depicted as a heatmap with bright yellow depicting the strongest binding, dark blue, no binding, and red, intermediate binding. The binding strength of each antibody directly corresponds to the abundance of the specific glycan epitope structure it recognizes. Amount of material recovered (mg/g AIR) from each sequential extraction shown at the top of each panel. Data are the mean of three biological replicates. Thin bars represent standard error. Letters denote statistical significance at α = 0.05.

Glycome profiles were generated as means from three saplings (biological replicates) per treatment (Figures 2–**4**). Differences in mean profiles among treatments and organs were clear, whereas there was little variation in individual profiles among biological replicates. Data for each biological replicate and a side-by-side comparison among organs are depicted in Supplementary Figure 1. Raw data are provided as means of the three biological replicates (Supplementary Table 2). Mean glucose equivalent carbohydrate contents for all cell wall extracts are shown in Supplementary Table 3.

### Variation in cell wall ultrastructure of loblolly pine organs in response to moisture stress

The glycome profiles of stem wood, roots, and needles indicated that cell wall ultrastructure responded to soil moisture treatments, especially in stem wood (Figures 2–**4**). The most obvious response was increased extractability of pectic backbone epitopes, those recognized by HG (homogalacturonan)-backbone 1 through 2 groups of mAbs, in the low compared to high soil moisture treatment, as evidenced by the higher abundance of these epitopes in the oxalate extracts (yellow dotted blocks in Figures 2–**4**). The increased extractability of pectic backbone epitopes in response to low soil moisture was most apparent in stem wood and roots, but also occurred to a lesser extent in needles. The oxalate extract of stem wood cell walls contained a higher abundance of rhamnogalacturonan-I (RG-I) backbone epitopes (another class of pectic backbone epitopes that are specifically recognized by RG-I backbone group of mAbs, yellow dotted blocks in Figure 2) in the low compared to high soil moisture treatment. Stem wood cell walls also exhibited increased abundance of pectic backbone epitopes (indicated by the increased binding of HG backbone-1 group of mAbs) in 1 M KOH (potassium hydroxide) and 4 M KOHPC (PC-post chlorite) extracts in the low compared to high soil moisture treatment. Further, the chlorite extract of stem wood exhibited an overall increase in the extractability of pectic backbone and pectic arabinogalactan epitopes (Figure 2 yellow and light blue dotted blocks, respectively) in the low compared to the high soil moisture treatment. Finally, stem wood cell walls exhibited a general increase in the amount of carbohydrate materials recovered (as denoted by the top bar graphs of Figure 2B) in both oxalate and carbonate extracts, which were enriched in pectic polysaccharides in the low compared to high soil moisture treatment. Where the optical density (OD 450–650 nm) differed >0.08, we provide statistical results (Supplementary Table 4).

### Cell walls differ among stem wood, roots, and needles of loblolly pine

Glycome profiles of well-watered loblolly pine stem wood, roots, and needles (Figures 2A, 3A, 4A) demonstrated that cell wall composition, integrity, and architecture differed among these organs. These differences were shown by the alterations in overall composition and extractability of non-cellulosic cell wall glycans. A difference was observed in the patterns of amounts of carbohydrate materials isolated during different extraction steps with the highest amount of material in 4 M KOHPC extracts from stem wood, chlorite and 4 M KOHPC extracts from roots, and carbonate extracts from needles. Compared to stem wood and roots, the amounts of carbohydrate materials extracted from needles were more evenly distributed; with the exception of the oxalate extract which showed a lower amount (see top bar graphs of (Figures 2A, 3A, 4A). These results, in general, demonstrate that overall extractability of cell wall components in loblolly pine stem wood, roots, and needles differed, which indicated differences in cell wall integrity and architecture among these organs. In general, glucose equivalent carbohydrate content was similar among treatments within organs except 4 M KOH, chlorite, and 4 M KOHPC in the stem wood where moisture stress resulted in higher carbohydrate contents.

Glycome profiles further indicated different abundances of cell wall glycan epitopes among corresponding extracts from different organs. Cell walls of needles showed a reduction of 1 M KOH extractible xyloglucan (XG) epitopes, especially those recognized by XG-4, XG-5, and XG-6 groups of mAbs, compared with the corresponding extracts from both stem wood and roots, which contained higher abundance of these epitopes (see 1 M KOH bars in Figures 2A, 3A, 4A). Cell walls of all three organs contained fucosylated xyloglucans (as indicated by the binding of FUC-XG group of mAbs) and their patterns of binding were similar among all organs with 1 M KOH, 4 M KOH, and 4 M KOHPC extracts exhibiting the highest abundance.

Extractability of xylan epitopes differed among organs. For instance, xylan epitopes recognized by the xylan-6 group of mAbs were low in abundance in all extracts from roots with the exception of 4 M KOHPC extract. However, these epitopes were abundant in 1 M KOH and 4 M KOHPC extracts from stem wood and 4 M KOH and 4 M KOHPC extracts from needles. Further, 1 M KOH extract from stem wood cell walls contained abundant xylan epitopes that are recognized by all dicot xylan-specific mAbs belonging to xylan-4 through xylan-7 groups of mAbs. However, 1 M KOH extracts from roots and needles predominantly contained only xylan epitopes recognized by xylan-5 and xylan-7 groups of mAbs. The 4 M KOH extract from needles was unique in that it contained abundant xylan epitopes that are recognized by all dicot xylan-specific mAbs belonging to xylan-4 through xylan-7 groups, which differed from the corresponding extracts from both stem wood and roots. Chlorite extracts from both stem wood and needles showed higher abundance of xylan epitopes (especially those recognized by xylan-5 and xylan-7 groups of mAbs) compared with extract from roots. These results may indicate that integration of xylan components through association with lignin in cell walls differs among loblolly pine organs.

Oxalate extracts from stem wood and needles contained acetylated glucomannan epitopes while root oxalate extracts contained only trace amounts of these epitopes. Pectic backbone epitopes (those recognized by HG backbone-1 through-2 and RG-I backbone groups of mAbs) were present in extracts from all three organs. However, the patterns of extractability varied. For example, carbonate extracts from roots contained lower abundance of rhamnogalacturonan-1 backbone epitopes compared to those from stem wood and needles. Again, reduced abundance of pectic backbone epitopes (those recognized by HG backbone-1 through-2 and RG-I backbone groups of mAbs) was noted in chlorite extract from roots in comparison to those from stem wood and needles. In general, a low abundance of pectic arabinogalactan epitopes (those recognized by RG-I/AG and AG-1 through AG-4 groups of mAbs) was evident in most extracts from all organs studied. However, there was variation across extracts from different organs. For example, certain arabinogalactan epitopes (especially those recognized by JIM11, MAC204, JIM20, JIM14, JIM12, CCRC-M133, and CCRC-M107) were present in higher abundance in 1 M KOH extracts from stem wood compared to extracts from roots and needle walls.

Subtle differences were also noted in carbonate and chlorite extracts in the case of certain epitopes recognized by some mAbs belonging to RG-Ic (CCRC-M56) and RG-I/AG (CCRC-M80 and CCRC-M79) groups, which showed a marginally higher abundance in extracts from needles compared to extracts from root and stem wood. Galactomannan epitopes (those recognized by the mAb, CCRC-M168 belonging to Galactomannan-2 group) were detected in all organs with extracts from needle cell walls exhibiting higher abundance. Where the optical density (OD 450–650 nm) differed >0.08, we provide statistical results (Supplementary Table 5).

## Discussion

### Variation in response to soil moisture availability

Our study, for the first time, reports organ-specific variation in the composition, integrity, and architecture of cell walls in trees and identifies specific modifications that occurred in cell walls in response to soil moisture. This study is also unique in that glycome profiling of pine needles has never before been reported. Although a glycome profiling study has previously been reported for loblolly pine (Pattathil et al., 2015), the analysis did not consider specificity to different organs or any abiotic stress. The purpose of including our physiological and growth data was to substantiate that the material used for glycome profiling was derived from plants that exhibited physiological responses to soil moisture availability that were both instantaneous (i.e., *A, E*, and *g*_*S*_,) and integrated over time (i.e., cumulative growth and leaf δ^13^C). Those results clearly demonstrated differences in physiological responses to soil moisture treatments; therefore, this discussion focuses on differences in glycome profiles in response to those treatments. Our hypothesis related to variation in cell wall ultrastructure in response to soil moisture availability, formulated based on information gained indirectly through previous proteomic and transcriptomic research, was largely supported by the glycome analysis conducted in this study. Specifically, we found that saplings grown in low soil moisture conditions exhibited enhanced extractability of pectic backbone epitopes from cell walls of their organs. Overall, these results demonstrate that soil moisture influences both the physiology and the functional structure and composition of the cell walls comprising the organs involved in water transport from roots through stem wood and leaves.

The strongest response of cell wall ultrastructure to soil moisture—and the most consistent across organs—was increased extractability of pectic backbone epitopes in the low soil moisture treatment. This treatment effect is indicated by the higher abundance of homogalacturonan and rhamnogalacturonan-1 backbone epitopes in the oxalate extract, suggesting that these components were more loosely integrated into the cell walls in saplings exposed to low soil moisture. The difference in extractability between saplings grown at high and low soil moisture was particularly large in stem wood and roots. Indeed, the role of cell wall components, such as pectins, in stress response has been well documented (Jarvis, 2009; Seifert and Blaukopf, 2010; Wolf and Greiner, 2012; Le Gall et al., 2015) and may suggest that loose binding of cell wall pectin components is important in stress response and/or signaling (Seifert and Blaukopf, 2010). For example, pectic polysaccharides such as PGAs (polygalacturonic acids) and OGAs (oligogalacturonic acids) have been implicated in biotic and abiotic stress signaling responses (Wolf and Greiner, 2012; Le Gall et al., 2015) and pectin-degrading enzymes have been shown to be down-regulated in response to moisture stress (Bray, 2004; Pandey et al., 2010). In fact, transgenic rice with increased pectin-degrading enzymes and, therefore, less pectins, was more susceptible to moisture stress than the wild type (Liu et al., 2014). The loosening of the pectic components in response to stress might trigger stress-signaling responses and thus initiate a cascade of stress-mitigating processes. Moreover, increases in cell wall elasticity, which may be related to drought tolerance (De Diego et al., 2013), have been observed under moisture stress conditions in avocado cultivars (Chartzoulakis et al., 2002), *Quercus* species (Saito and Terashima, 2004) and *Pinus radiata* genotypes (De Diego et al., 2013). These stress-induced alterations in cell wall elasticity may involve “cell wall loosening” processes resulting from the rearrangement of cell wall structural components including major matrix wall polysaccharides such as pectins and hemicelluloses.

Variation in cell wall ultrastructure may have large ramifications for physiological function. In the cell wall, pectins form a hydrophobic gel phase that prevents the collapse of the cellulosic network (Jarvis, 1992). It has therefore been suggested that the pectic components of the cell wall are involved in the important function of responding to low cell turgor (Jarvis, 1984, 1992). Mechanical evidence from expanding pollen tubes of *Solanum chacoense* (Parre and Geitmann, 2005) demonstrated that both the amount of pectins and the degree of esterification were important factors in determining cell wall stiffness and other cytomechanic properties. Specifically, an increase in the abundance of pectins was associated with increased resistance to tensile stress. The increased pectin content observed under moisture stress in stem wood in our study may have allowed xylem conduits to withstand higher tension of the water column or for tension to be more evenly distributed along the xylem conduit. It has further been speculated that pectins, as components of hydrogels, play a role in modulating the flow of water and/or air at the pit and torus structures at tracheid junctions (Zwieniecki et al., 2001).

Under moisture stress, all organs showed the general response of increased pectic extractability, with stem wood showing the greatest increase. In addition, compared to other organs, moisture-stressed stem wood exhibited a higher abundance of pectic arabinogalactan epitopes (indicated by increased binding of some of RG-I/AG groups of mAbs) and arabinogalactan epitopes (indicated by increased binding of some of AG groups of mAbs) in the chlorite extracts, hinting at higher lignin-cell wall carbohydrate associations in this organ. These results suggest that cell walls of the stem wood were more involved in moisture stress response processes than were cell walls of roots or needles. Although all organs we examined are involved in water transport, the stem transports a much larger quantity of water than does any individual root or needle, so it follows that responses of cell walls to moisture stress might be most pronounced in this organ. In addition, the stem is a perennial organ as opposed to an ephemeral organ like the needles and fine roots we investigated. In addition to the functional role of water transport, the perennial stem also provides critical structural support. Perhaps perennial tissues exhibit a higher capacity for modification of their cell wall ultrastructure than do ephemeral tissues because ephemeral tissues are routinely replaced, whereas perennial tissues must be maintained for a longer duration. It is plausible that early exposure to moisture stress may result in cell wall ultrastructure in the stem wood that is optimized for moisture stress since these cells will ultimately die and consequently be unable to further modify their ultrastructure; however, they will remain a critical component of water transport for several years and provide structural support for the life of the tree. Given the different responses we observed between perennial and ephemeral organs and their functional roles, we might expect that coarse roots, tap roots, and branches would respond more similarly to stems than to ephemeral fine roots or needles.

It would be beneficial if future research on the effects of water stress on cell wall ultrastructure focused on additional species and functional groups. Cell wall ultrastructure may respond to water stress less in species with broad site requirements compared to those with narrow site requirements (Aubrey et al., 2012) or less in xeric-site species compared to mesic-site species. Also, the plasticity of cell wall ultrastructure is not well understood in plants. It is possible that the effects of moisture stress on cell wall ultrastructure could be reversed if the stress was removed. If this was the case, it would be important to know how rapidly and the degree to which these changes could occur. It is also important to consider that the responses to moisture stress observed here may have been a direct response to the stress, or an indirect response due to the effect of water stress on plant size and development. The imposed drought stress resulted in smaller plants that may have been developmentally different than the control plants. Research studies designed to separate direct and indirect effects of water stress on cell wall ultrastructure are needed to address this issue.

### Variation among organs

To our knowledge, no studies have elucidated variation among organs of loblolly pine in structure and composition of the major non-cellulosic cell wall matrix glycans or their dynamics. Moreover, only a handful of studies (mostly pertaining to wood formation) have been conducted in loblolly pine on the identification, functional annotation, or characterization of cell wall biosynthetic genes (Zhang et al., 2000; Whetten et al., 2001; Kirst et al., 2003; Nairn et al., 2008). However, studies have been conducted in loblolly pine on wood cell wall physical properties (Cramer et al., 2005; Zelinka et al., 2012) and ultrastructural features such as wood cell wall structural proteins (Bao et al., 1992), distribution of certain non-cellulosic cell wall glycans such as (1–4)-β-galactans, (1–3)-β-glucans, arabinogalactan proteins (AGPs), and xylans (Altaner et al., 2010), and lignin or lignin-polysaccharide linkages (Minor, 1982; Ralph et al., 1997). In *Pinus radiata*, a closely related species, localization studies have been conducted on distribution of non-cellulosic glycans in wood tissues using a collection of cell wall glycan directed antibodies (Donaldson and Knox, 2012). Transcriptomic studies have further demonstrated large differences in gene expression in moisture stressed roots and needles of loblolly pine (Lorenz et al., 2006, 2011).

Overall extractability, based on the amount of carbohydrate materials recovered per extraction step, differed among organs indicating differences in cell wall composition, integrity, and architecture. In all organs studied, hemicellulose components such as xyloglucan and xylan were detected, shown as significant binding of mAbs specific to epitopes of these glycans across extracts. These results demonstrated that cell walls of all organs contained xyloglucans with fucosylated and non-fucosylated structural regions. In addition, xylan with unsubstituted (homoxylan) and substituted (glucuronoarabinoxylans or arabinoxylans) structural features was indicated by the binding of xylan-4 through xylan-7 groups of mAbs (Schmidt et al., 2015). Pectic backbone polysaccharides were also detected in all organs along with the presence of pectic arabinogalactans (pectic AG) and arabinogalactans (AG). Glycome profiling also demonstrated the presence of other important glycans that are characteristic of gymnosperms, namely, glucomannan and galactomannan.

Although the cell wall composition of all three organs consisted of a similar broad suite of non-cellulosic glycans, the glycome profiles demonstrated that the integration of these glycans into the final wall structure differed among organs, as indicated by the different patterns of glycan extractability among organs. Obvious examples for these variations in extractability patterns among organs are exhibited by the xyloglucans and xylans. For instance, compared with stem wood and roots, needles showed a significantly reduced abundance of non-fucosylated xyloglucan epitopes (recognized by non-fuc xyloglucans-3 through non-fuc xyloglucans-6 groups of mAbs) in 1 M KOH extracts. In the case of xylan, extractability patterns were substantially different among organs. In roots—but not stem wood or needles—xylan epitopes recognized by xylan-6 groups of mAbs were not detected until the last extraction step, 4 M KOHPC. Further, chlorite extract from roots contained a reduced abundance of xylan and pectic arabionogalactan epitopes (potentially due to reduced presence of lignin) compared to stem wood and needles. Overall, these results emphasize that cell wall composition, integrity, and architecture varied significantly among loblolly pine organs. The relative differences in cell wall ultrastructure between secondary and primary cell walls may have influenced our comparisons among organ types and this effect should be considered in future analyses.

### Conclusion and future perspectives

Overall, our results indicate that loblolly pine organs modify cell wall ultrastructure in response to moisture stress and that cell walls of stem wood respond more strongly than cell walls of roots or needles. While our results do not explain changes in physiological function that may be elicited through modifications to cell wall ultrastructure, they do offer a direction for future research efforts to focus more closely on the coupling of cell wall ultrastructure (i.e., form) and physiological function. In addition to the potential physiological adjustments that may occur as a result of moisture stress-related modifications to cell wall ultrastructure, there are also potential ecological and economic implications. For example, moisture stress may influence cell wall ultrastructure in ways that ultimately influence wood quality and value. Likewise, differences in cell wall ultrastructure may also influence the recalcitrance of woody material and thus, the residence time for decomposition in a natural ecosystem or the ease with which enzymatic or chemical degradation can convert woody material to a biofuel. The specific objective of this study was to investigate variations in cell wall ultrastructure of loblolly pine organs as a function of moisture availability. Based on the findings obtained here, future investigations should seek to understand how specific cell wall structures/linkages of different cell types are influenced by moisture availability. Toward these objectives, we intend to conduct wet-chemistry and immunolocalization studies in the future. Now that we have directly demonstrated modifications in cell wall ultrastructure in response to moisture stress, we would expect responses to other abiotic stresses, and suggest that imposing these stresses may provide a path toward eventually being able to manipulate cell wall structural characteristics to improve both the physiological function of plants as well as the quality of products that we derive from them. For example, the cell wall modifications in response to moisture stress that we describe here could be readily compared and correlated to gene expression responses. Such multi “omic” approaches could assist in identifying the genes associated with cell wall characteristics that are either up- or down-regulated in response to abiotic stress. Several of these differentially expressed genes might play important roles in combating stress in plants. Supporting this notion, previous studies that elucidated the switching on of genes in response to moisture stress in pines observed differential regulation of several genes associated with cell wall characteristics (Eveno et al., 2008; Lorenz et al., 2011). Our study advances questions regarding regulation of specific cell wall biosynthetic or wall-modifying genes and their effects on moisture stress resistance. For instance, one hypothesis that merits further investigation is whether up-regulation of pectin biosynthetic genes can provide plants with a better signaling mechanism and thus better stress resistance and survival.

## Author contributions

SP, MI, MM, RT, and DA planned and designed the experiments, interpreted the data, and wrote manuscript. SP, MI, OV, and SK performed all the experiments and conducted analysis of data.

## Funding

This project was supported by Agriculture and Food Research Initiative Competitive Grant no. 2011-67009-30065 and 2013-67009-21405 from the USDA National Institute of Food and Agriculture. We also acknowledge Bio Energy Science Center (BESC) administered by Oak Ridge National Laboratory and funded by a grant (DE-AC05-00OR22725) from the Office of Biological and Environmental Research, Office of Science, United States, Department of Energy. The generation of the CCRC series of plant cell wall glycan-directed monoclonal antibodies used in this work was supported by the NSF Plant Genome Program (DBI-0421683 and IOS-0923992).

### Conflict of interest statement

The authors declare that the research was conducted in the absence of any commercial or financial relationships that could be construed as a potential conflict of interest.
